# Diagnostic tools for neurosyphilis: a systematic review

**DOI:** 10.1186/s12879-021-06264-8

**Published:** 2021-06-14

**Authors:** Gustavo Henrique Pereira Boog, João Vitor Ziroldo Lopes, João Vitor Mahler, Marina Solti, Lucas Tokio Kawahara, Andre Kakinoki Teng, João Victor Taba Munhoz, Anna S. Levin

**Affiliations:** 1 Faculdade de Medicina, Universidade de Sao Paulo, Sao Paulo, Brazil; 2 Infection Control Department, Hospital das Clinicas, Faculdade de Medicina, Universidade de Sao Paulo, Sao Paulo, Brazil

**Keywords:** Neurosyphilis, Syphilis, Cerebrospinal fluid, Diagnosis, Diagnostic tests, Accuracy

## Abstract

**Purpose:**

Increasing incidences of syphilis highlight the preoccupation with the occurrence of neurosyphilis. This study aimed to understand the current diagnostic tools and their performance to detect neurosyphilis, including new technologies and the variety of existing methods.

**Methods:**

We searched databases to select articles that reported neurosyphilis diagnostic methods and assessed their accuracy, presenting sensitivity and specificity values. Information was synthesized in tables. The risk of bias was examined using the Cochrane Handbook for Systematic Reviews of Diagnostic Test Accuracy recommendations.

**Results:**

Fourteen studies were included. The main finding was a remarkable diversity of tests, which had varied purposes, techniques, and evaluation methodologies. There was no uniform criterion or gold standard to define neurosyphilis. The current basis for its diagnosis is clinical suspicion and cerebrospinal fluid analysis. There are new promising tests such as PCR tests and chemokine measurement assays.

**Conclusions:**

The diagnosis of neurosyphilis is still a challenge, despite the variety of existing and developing tests. We believe that the multiplicity of reference standards adopted as criteria for diagnosis reveals the imprecision of the current definitions of neurosyphilis. An important next step for the scientific community is to create a universally accepted diagnostic definition for this disease.

**Supplementary Information:**

The online version contains supplementary material available at 10.1186/s12879-021-06264-8.

## Introduction

Neurosyphilis is a condition that has challenged physicians for centuries. The invasion of the Central Nervous System by *Treponema pallidum* subspecies pallidum can result in protean symptoms ranging from vasculitis, stroke, dementia, and meningitis to completely asymptomatic presentations [1]. Diagnostic tools have a far from ideal performance and thus a high degree of suspicion of the diagnosis is needed to properly identify the condition [2, 3]. None of the existing tests can be considered a good and applicable gold standard, and until now there is no consensus regarding diagnostic criteria for this disease [4].

The incidence of acquired syphilis has been rising in several countries [5–8]. Thus, knowledge of the performance capabilities and limitations of diagnostic tests is crucial for clinicians to properly diagnose and treat those afflicted by this morbid complication of untreated syphilis. In this respect, there are diagnostic accuracy systematic reviews that assessed certain tools. A review evaluated polymerase chain reaction (PCR) techniques and found out that their sensitivity is low compared to cerebrospinal fluid (CSF) serological assays, despite the limitation of not having a good gold standard [9]. Another study showed that CSF treponemal-specific antibody tests have a variable performance and a dependent relation to the prevalence (pre-test probability) of neurosyphilis [10]. Here we conducted a systematic review aiming to investigate the performance and limitations of all the current diagnostic tests assessed in the most recent literature.

## Materials and methods

This systematic review was performed based on the Preferred Reporting Items for a Systematic Review and Meta-analysis of Diagnostic Test Accuracy Studies: The PRISMA-DTA statement [11]. It was registered on the International Prospective Register of Systematic Reviews (PROSPERO; available from https://www.crd.york.ac.uk/prospero/display_record.php? ID=CRD42020181755) [12].

### Search strategy

Systematic literature review based on online search in PubMed from National Center for Biotechnology (NCBI), Scientific Electronic Library Online (SciELO) and Embase databases was done on 18th April 2020. The following terms were used in the search engine for any match in articles: ((Syphilis) OR (Treponema pallidum)) AND ((Neurosyphilis) OR (Tabes Dorsalis) OR (Central Nervous System)) AND (Diagnosis). We limited the search to studies published from 2015 to 2020. Duplicates were deleted, using the Endnote (Clarivate Analytics) reference engine.

### Inclusion and exclusion criteria

We selected papers that reported neurosyphilis diagnostic methods and strategies for patients with *Treponema pallidum* infection, regardless of the clinical presentation, that assessed their performance in comparison with a gold standard. We included only observational and diagnostic test studies. Clinical trials, reviews, case reports, research protocols, and presentations at conferences were not considered.

We excluded papers that were unpublished, inaccessible, or incomplete. If the article did not present a diagnostic method and its performance, it was also excluded from this review.

### Assessment of risk of Bias

The analysis of the methodological quality of the studies was made using the recommendations of the Cochrane Handbook for Systematic Reviews of Diagnostic Test Accuracy [13], which is based on the QUADAS (Quality Assessment of Diagnostic Accuracy Studies) instrument [14]. This methodological quality assessment was presented as a summary figure and a graph figure. The instrument can be found in the web-only supplementary figure (Online Resource 1).

### Study selection

After the deletion of duplicates, we screened the papers by title. Next, each abstract was assessed by two independent authors. The full text was evaluated for any potentially relevant study and reviewed by two authors to determine if they met the eligibility criteria. A third author was asked to analyze in case of discordance.

### Data extraction

From the included articles, we used the Google Sheets application (Google INC.) to organize extracted data regarding the study design and limitations (diagnostic test study, case-control, cross-sectional, cohort); the sample characteristics (age distribution, sex, HIV-positivity), the diagnostic method used, the gold standard used, and the evaluation of performance (sensitivity and specificity values) of the test. Categorical information also was collected by two separated authors. The data organized in Google Sheets was subsequently summarized in tables.

## Results

### Records

**Fig. 1:**
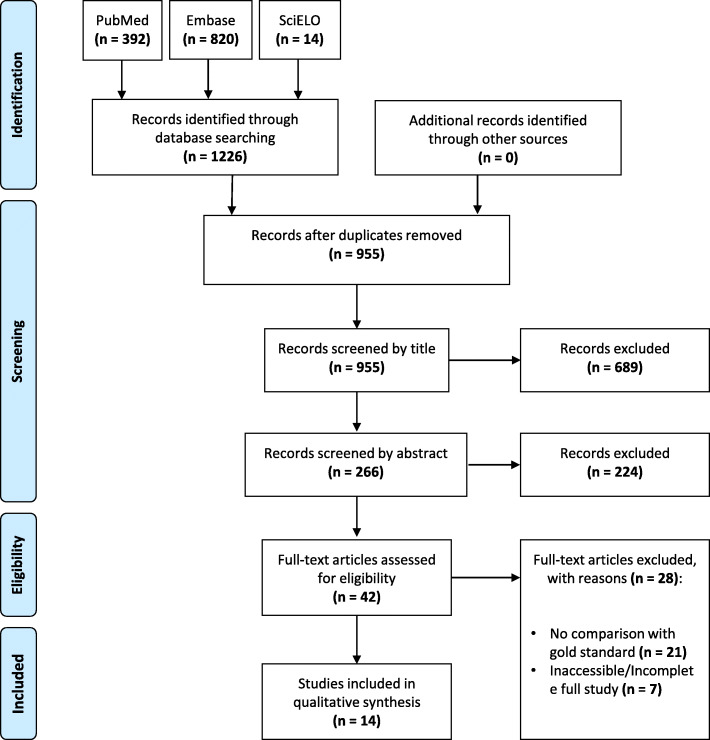
PRISMA (Preferred Reporting Items for Systematic Reviews and Meta-Analyses) flow chart, representing the selection of studies

Fourteen studies were included from the 1226 papers found in our initial search. The steps of our selection process are presented in Fig. 1.

Regarding the study design, ten were studies of diagnostic tests, three were cross-sectional studies, and one was case-control. Information about the articles and their sample characteristics can be seen in the web-only supplementary table (Online Resource 2).

### Bias assessment

**Fig. 2:**
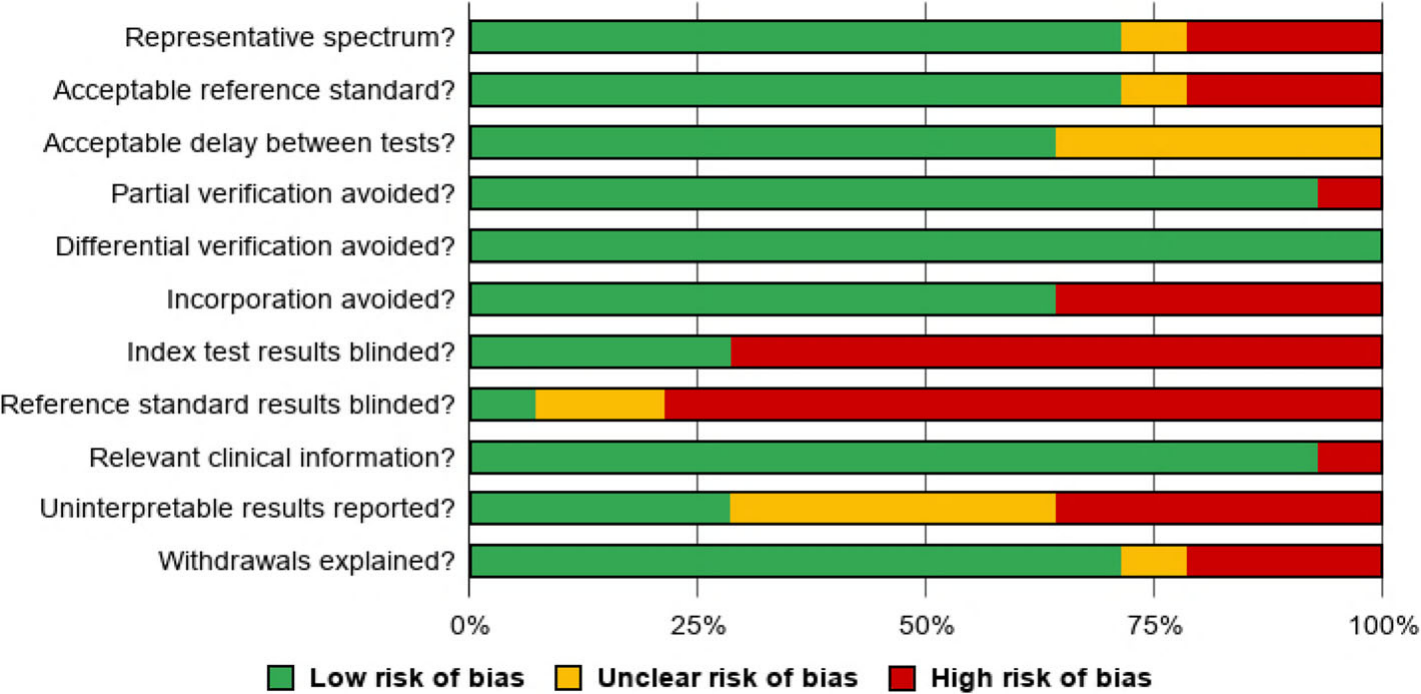
Methodological quality graph: proportions of low, unclear, and high risk of bias of the studies included in this review, according to the Cochrane Handbook for Systematic Reviews of Diagnostic Test Accuracy recommendations [13]

**Fig. 3:**
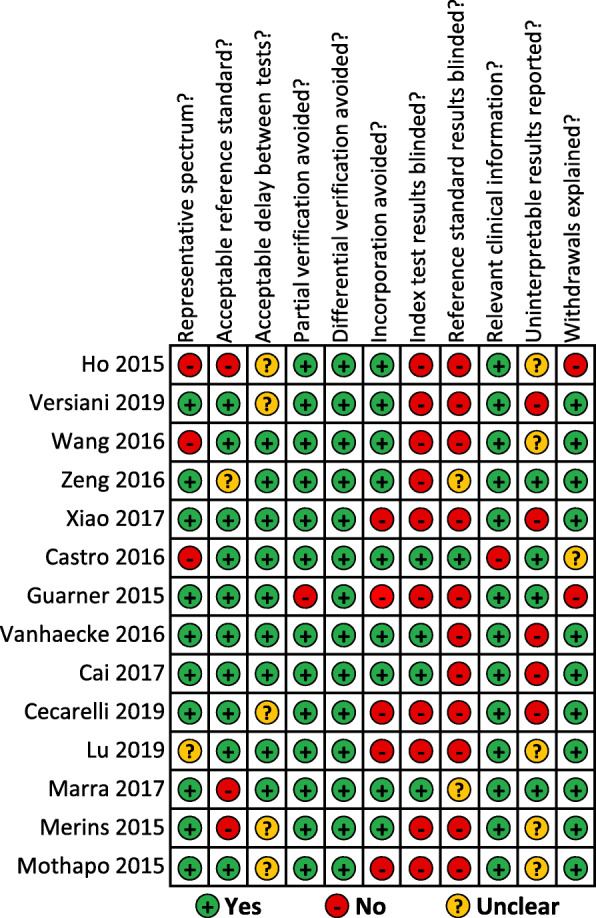
Methodological quality summary for risk of bias for all studies. Based on the Cochrane Handbook for Systematic Reviews of Diagnostic Test Accuracy recommendations [13]

The results of the methodological assessment are described in Fig. 2, which shows the overall quality of the 14 studies included. The individual analysis for each study can be seen in Fig. 3.

### Diagnostic tests and their performance

The main findings of our review with 14 studies are presented in Table 1. Gold standards used to evaluate the diagnostic methods studied varied widely. Some papers used more than one definition of neurosyphilis.

**Table 1:** Main Results. Diagnostic methods for neurosyphilis and their performance

Type of diagnostic method	Test	Performance (%)		Gold standard used to define neurosyphilis	Sample size (n)			HIV (+) Status (%)	Commentaries	Ref.
		Sensitivity	Specificity		NS (+)	NS (−)	Control			
Clinical	Neurological symptoms	46%	33%	CSF-VDRL (+) OR CSF-VDRL (−) AND: **a.** CSF-WBC **>** 5 cells/μL OR **b.** CSF-protein **>** 450 mg/L OR **c.** Neurological symptoms without other known cause	50	50	0	0%	Case-control study that evaluates the usefulness of TPPA as a diagnostic tool, either alone or associated with other criteria. Consider this test when there is clinical suspicion and negative CSF-VDRL. Possible selection bias due to retrospective inclusion of patients who underwent lumbar puncture. Another limitation is that some of the diagnostic tests evaluated are also included in the gold standard employed by the study, thus generating performance analysis confusion.	15
	Neurological symptoms AND CSF-protein **>** 497 mg/L AND CSF-WBC **>** 3 cells/μL	89%	98%							
	Neurological symptoms AND CSF-protein **>** 497 mg/L AND CSF-WBC **>** 3 cells/μL AND CSF-TPPA **>** 1:160	92%	40%							
Laboratory (CSF)	CSF-protein **>** 497 mg/L	54%	85%							
	CSF-WBC **>** 3 cells/μL	48%	82%							
	CSF-TPPA **>** 1:160	90%	84%							
	CSF-TPPA **≥** 1:80	95% (**I**) 76% (**II**) 68% (**III**)	–	Three gold standards used: **I.** CSF-VDRL (+) **II.** *T. pallidum* RT-PCR (+) **III.** New loss of vision or hearing	105	86	0	78%	Evaluated the accuracy of CSF-TPPA, by comparing it with the sensitivity of CSF-FTA-ABS in a first group (*n* = 191), and with the specificity of VDRL in a second group (*n* = 380). A reported limitation is that the sample does not represent the population of patients with syphilis. Most patients were HIV (+). We considered that the clinical definition (**III**) of NS lacked the precision to evaluate any diagnostic test.	16
	CSF-TPPA **≥** 1:640	–	97% (**I**) 94% (**II**) 93% (**III**)		120	260	0			
	Reactive CSF-FTA-ABS	89%	22%	ITPA index (TPPA CSF/serum ratio) **>** 2 AND: **a.** CSF-FTA-ABS (+) OR **b.** CSF-RPR **>** 1:1 OR **c.** CSF pleocytosis **>** 4 cells/μL OR **d.** CSF-protein **>** 500 mg/L OR **e.** Albumin quotient (CSF/serum) **>** 7.8	38	29	0	52%	The study correlates pleocytosis and albumin quotient with NS (+) (independent of HIV co-infection). Highlights the importance of lumbar puncture in diagnosing asymptomatic patients, especially in the HIV (+) population. There is a possible selection bias due to the high clinical suspicion in the patient’s inclusion in the study.	17
	Reactive CSF-RPR	21%	97%							
	Reactive CSF-RPR	100%	100%	Clinical suspicion, serological treponemal reactive test AND **a.** CSF-VDRL (+) OR **b**. CSF-WBC > 5 cells/μL OR **c.** CSF-protein > 450 mg/L **NS confirmed** if the CSF-VDRL is reactive. **NS suspected** if it is nonreactive.	21 (confir-med NS)	49 (sus-pected NS)	50	Not reported	Compared the performance of different treponemal tests (RPR and USR) with each other using CSF-VDRL as a standard. There was perfect qualitative agreement (kappa value = 1) between evaluated tests and VDRL; sensitivity and specificity were both 100%. These values should be understood as evidence of diagnostic equivalence between these tests and the standard (VDRL), which has its own limitations. Considering this NS definition, USR and RPR are as good as VDRL to differentiate between confirmed and suspected NS. The study did not report HIV status and only included patients with neurological symptoms.	18
	Reactive CSF-USR	100%	100%							
	Reactive CSF-VDRL	54% (**I**)69% (**II**)	75% (**I**)73% (**II**)	The authors mentioned CSF-VDRL as gold standard (^a^), but used the following definitions for the performance analysis: **I.** CSF-WBC **>** 20 cells/μL (regardless of other variables) **II.** Vision or hearing loss (regardless of other variables)	54 (^a^) 152 (**I**) 145 (**II**)	163 (^a^) 65 (**I**) 72 (**II**)	0	70%	Concludes that the specificity of CSF-SYPHICHECK with cutoff, and sensitivity without cutoff perform similar to CSF-VDRL and remarks that titers rapidly normalize after treatment. Reports impaired patient humoral response due to high prevalence of HIV coinfection. There was no comparison with healthy or control patients. Definitions used for evaluation were not justified with references and we considered them to be imprecise for test performance evaluation.	19
	Reactive CSF-FTA-ABS	70% (**I**) 81% (**II**)	54% (**I**)51% (**II**)							
	CSF-SYPHICHECK	62% (**I**)64% (**II**)	57% (**I**)53% (**II**)							
	CSF-SYPHICHECK **≥** 1:4	37% (**I**)44% (**II**)	81% (**I**)79% (**II**)							
	CSF-VDRL	85%	100%	**a.** Two reactive/positive tests (regardless if treponemal or nontreponemal) OR **b.** Reactive PCR.	18	0	14	38%	Among study limitations were the small sample size and the fact that the tests being evaluated were used as diagnostic criteria for NS (+), which increased its accuracy. Not all cases were tested with all methods due to the small volume of some specimens.	20
	CSF-TREPSURE	92%	100%							
	CSF-MAXISYPH	100%	100%							
	CSF-INNO-LIA	92%	100%							
	CSF-TPPA	83%	100%							
Laboratory (blood)	RPR 1:4	77%	80%	**I. Confirmed NS:** CSF-RPR (+) OR **II. Probable NS:** Syphilis of any stage with: **a.** CSF-protein **>** 500 mg/L OR CSF-WBC **>** 10 cells/μL (without another cause) AND **b.** Signs/symptoms consistent with NS (without another cause).	191	179	0	0%	Test performances were evaluated for NS (+) general detection (I OR II being the exposed values) and discriminating between confirmed (I) and probable (II), with a better accuracy being described for (I). A multivariate analysis found another biomarker, plasmatic CK-MB. The study included only HIV (−) patients with neurological symptoms, without control groups NS (−) or asymptomatic patients. RPR was used as the gold standard, which differs from most studies analyzed in this review, which used CSF-VDRL.	21
	TPPA **≥** 1:2560	83%	83%							
	RPR 1:2 OR TPPA **≥** 1:1280	96%	46%							
	RPR ≥ 1:16	32%	88%	**Asymptomatic NS:** **a.** No neurologic symptoms/signs AND **b.** CSF-RPR (+) OR **c.** WBC **>** 5 cells/μL OR **d.** CSF-protein **>** 450 mg/dL	139	263	0	0%	The sample included syphilis patients with persistent RPR titles after treatment. ANS was most frequent between ages 51–60 years, and the best cutoff value was 1:16. This study recommended lumbar puncture in patients with persistent RPR titles. Study limitations: the absence of HIV (+) population; patient’s outcome was not reported.	22
	RPR 1:32	67%	59%	**Asymptomatic NS:** **a.** CSF-RPR (+) OR **b.** CSF-WBC **>** 20 cells/μL AND CSF-TPPA **>** 1:640	12	19	0	100%	This study has a small sample size and restricted population characteristics (only latent syphilis, HIV (+), and asymptomatic patients). Uses RPR as diagnostic criteria, possibly interfering with the reported specificity/sensibility values.	23
	CD4 350	75%	82%							
	RPR 1/32 AND CD4 350	50%	67%							
Molecular	TP 47 PCR	76%	87%	CSF-TPHA/FTA-ABS (+) AND **a.** CSF-WBC **>** 5–10 cells/μL OR **b.** CSF-VDRL/RPR (+)	33	91	0	Mostly positive	Addresses PCR as a promising technique for NS diagnosis. The majority of the patients presented with latent syphilis. Study limitations: small sample size; no differentiation between latent syphilis stages (which interferes in the differentiation between late and early NS/meningitis); patient outcome not reported.	24
	POL A PCR	70%	92%							
	TPP 47 Nested PCR	42%	97%	Mentioned CSF-VDRL as gold standard, but used the following definitions for the analysis: **a.** Serological reactive non-treponemal and treponemal tests AND; **b.** Signs/symptoms AND; **c.** CSF abnormalities such as VDRL (+), FTA-ABS (+), elevated WBC, elevated proteins)	40	0	0	45%	Study considerations valid only for symptomatic patients (exclusion of patients without ophthalmic and neurologic symptoms). The study tested Nested PCR in samples of patients with confirmed NS according to the gold standard used. The study describes problems with sample preservation that could affect sensitivity. CMV coinfection was a confusion factor present.	25
Immunological biomarkers	CSF-CXCL13 **>** 256.4 pg/mL	85% 82% (ANS)	89% 88% (ANS)	**Confirmed NS**: CSF-VDRL (+) AND CSF-TPPA (+) **Presumed NS**: CSF-VDRL (−), CSF-TPPA (+), AND: **a**. CSF-WBC **>** 8 cells/μL or CSF-protein **>** 450 mg/L without another cause OR **b**. Signs/symptoms consistent with NS without another cause	191	123	92	0%	Chemokine levels were useful for patient follow-up (decreased after treatment). They may change due to other inflammatory conditions and previous treatments/medications. Not useful for HIV co-infection. Control serum and CSF samples were from different individuals.	26
	CSF-CXCL8 **>** 48.1 pg/mL	79% 71% (ANS)	90% 89% (ANS)							
	CSF-CXCL10 **>** 163.1 pg/mL	80% 69% (ANS)	91% 90% (ANS)							
	CXCL13 (CSF/serum) **>** 4.36	87% 83% (ANS)	99% 99% (ANS)							
	CXCL8 (CSF/serum) **>** 10.3	79% 68% (ANS)	73% 72% (ANS)							
	CXCL10 (CSF/serum) **>** 1.02	86% 77% (ANS)	92% 93%(ANS)							
	CSF-CXCL13 **>** 76.3 pg/mL	50%	90%	CSF-RPR (+)	16	87	0	54%	The study is limited by the lack of clinical data about previous patient’s treatment, and by the sole inclusion of patients that underwent lumbar puncture. CXCL13 added more diagnostic value to RPR when evaluating patients with HIV co-infection. There is a possible classification bias, as the gold standard disregards CSF abnormalities such as protein and WBC count.	27
	CSF-CXCL13 **>** 4.87 pg/mL	80%	81%	Not described in the article, just referenced [15]: Syphilis positive serologies AND **a.** CSF-VDRL/RPR (+) OR **b.** CSF-TPPA (+); an otherwise unexplained neurologic manifestation consistent with NS; CSF-proteins **>** 50 mg/dL or CSF-WBCs **>** 10 cells/μL	40	57	49	0%	No difference was reported for different clinical manifestations. There was evidence of intrathecal CXCL13 production. Controls did not undergo lumbar puncture, limiting comparisons.	28
	Quotient ^a^ **>** 2.408	88%	69%							

Main results of the 14 studies that evaluated diagnostic tests and criteria for neurosyphilis and their performance. *Abbreviations*: *NS* Neurosyphilis, *ANS* Asymptomatic neurosyphilis, *NS (+)* Positive neurosyphilis diagnosis, *NS (−)* Negative neurosyphilis diagnosis, *HIV* Human immunodeficiency virus, *CSF* Cerebrospinal fluid, *WBC* White blood cells, *VDRL* Venereal disease research laboratory, *RPR* Rapid plasma reagin, *USR* Unheated serum reagin, *TPPA* T. pallidum particle agglutination, *TPHA* T. pallidum hemagglutination, *FTA-ABS* Fluorescent treponemal antibody absorption, *PCR* Polymerase chain reaction, RT-PCR Reverse transcriptase polymerase chain reaction, *CMV* Cytomegalovirus, *CXCL* Chemokine CXC ligand

^a^ *Quotient = (CSF-CXCL13 / CSF-albumin) / (Serum-CXCL13 / Serum-albumin)*

The tests assessed in the included studies were methodologically very different. We grouped them in clinical (if they contained neurological symptoms or signs), laboratory (CSF or blood), molecular (PCR techniques), and immunological (chemokines levels).

The sample characteristics were not homogeneous. Some studies included only negative or HIV-positive patients or both; only symptomatic or asymptomatic or both. Most samples were predominantly composed of men, and the median/mean age varied from 33 to 53 years. Information regarding characteristics of each specific study (design, year, and country of publication) and sample (sex and age distribution) is summarised in the web-only supplementary table (Online Resource 2).

## Discussion

Considering the variety of tests and the incorporation of new technologies in clinical practice, we conducted this literature review aiming to understand what are the current and potential diagnostic methods for neurosyphilis and how they perform. The primary finding of our study was a remarkable diversity of tests, which had different purposes (diagnostic confirmation, screening), varied techniques (clinical signs/symptoms, serological analysis, CSF assessment), and a heterogeneous evaluation methodology (including or not HIV-positive individuals, including or not asymptomatic patients, comparing or not with controls, etc.). Most of the articles studied CSF alterations, measuring cells, proteins, treponemal and nontreponemal antibodies [16–20], or applied new immunological/biomolecular techniques [21–25]. Three papers assessed the significance of blood parameters to distinguish between NS+ and NS- [26–28], and only one considered clinical signs or symptoms in the investigation [29].

Among all the diagnostic tests for neurosyphilis, CSF-VDRL and CSF-RPR stand out. Both exams were considered as gold standards to confirm the diagnosis in most studies. However, there are important limitations to this choice: they are operator-dependent and have low sensitivity. Particularly, there are reports of groups of patients that have compatible clinical symptoms, positive treponemal test in blood and CSF, respond to penicillin treatment but still show negative CSF-VDRL/CSF-RPR [30]**.** This contributes to the great heterogeneity of classifications and patient selection for the studies, making it difficult to determine their biological and clinical implications.

The laboratory diagnosis is of utmost importance for NS. Currently, clinical suspicion of NS should prompt serum VDRL and FTA-ABS examination. Lumbar puncture is recommended for patients with neurological, otologic, or ocular symptoms, regardless of syphilis stage, including cases of treatment failure (patients with previous syphilis diagnosis and persistent high titer of serum VDRL despite adequate treatment) [31]. The current laboratory recommendation for NS diagnosis includes CSF analysis with non-treponemal tests such as VDRL or RPR (in the absence of CSF-VDRL), and with treponemal tests such as FTA-ABS, alongside CSF cellularity and protein levels. However, there are important limitations, as CSF non-treponemal tests are not sensitive enough and do not eliminate the possibility of NS in case of negative results [32]. On the other hand, CSF treponemal tests are more specific but less sensitive, so they do not confirm the diagnosis but can exclude it. Finally, the hypercellularity and elevated protein levels can support the diagnosis in the presence of a negative non-treponemal CSF test and warrant empiric treatment. That being said, the low sensitivity of CSF-VDRL is the most significant limitation, presenting a low negative predictive value.

Another challenge is regarding diagnosis in asymptomatic patients, investigated with usual tests for the hypothesis and diagnosis of neurosyphilis: FTA-ABS and RPR. Laboratory parameters (such as increased protein and leukocyte levels or even positive RPR) do not offer a significant statistical gain to confirm the disease, but, if not altered, they moderately reduce the individual’s chance of having neurosyphilis.

In addition to this classic analysis of the CSF, new technologies have emerged: biomolecular tests and chemokine measurement. The polymerase chain reaction (PCR) is relevant given its increased specificity - with the lowest value of 86.8% - associated with a sensitivity of intermediate values compared to the other tests - between 42 and 75.8%. When applied to a clinical setting these tests show a moderate likelihood ratio increase both in positive and negative diagnosis. Diagnostic evaluation with the use of genetic material from infectious agents is a common practice in the microbiological clinic [33–35]. However, given the heterogeneity of studies, the population involved, and diagnostic criteria, the routine use of the PCR technique for diagnosing neurosyphilis is not yet fully implemented. Additionally, there are other limitations such as availability and cost that may hinder its use.

The measurement of chemokines shows higher sensitivity values - ranging from 50 to 88% - but also maintains high specificity values - which ranged between 69 and 99%. The relevance of this technique consists in the possibility of identifying specific changes in the CNS, distinguishing infectious and non-infectious stress patterns. Additionally, considering more precise immune responses depending on the agent, the immune profile present in the CSF may allow, in the future, a diagnosis based on the chemokine profile and not agent identification or specific antibodies for it. However, being a recent and not fully explored technique, it has challenges, such as its accessibility, demanding specific ELISA kits, and, still, the need for having a well-described chemokine and cytokine CSF profile in health and different diseases.

Because current tests lack sensitivity, new research has been exploring novel CSF biomarkers and their potential to aid in the diagnosis or exclusion of NS. For instance, myeloid and microglial activation markers such as MIF (Macrophage migration inhibitory factor) and sTREM2 (soluble Triggering receptor expressed on myeloid cells 2) have been reported to be differentially expressed in the CSF of patients with NS and have emerged as promising tools for establishing a diagnosis, particularly in the setting where non-treponemal tests are negative but there is high clinical suspicion [36, 37]. Furthermore, Zhang et al. reported that several CSF proteins such as neurogranin, BACE1, and Tau are increased in patients with Alzheimer’s Disease in comparison to those with NS, which may be useful in the setting of patients with cognitive decline and a past history of syphilis [38]. IL-10 has also been reported to be useful in increasing the likelihood of NS [39]. These findings are exciting and may provide clinicians with new biomarkers to assist in the confirmation or exclusion of NS in the future, however, more studies in larger populations should be conducted.

**Fig. 4:**
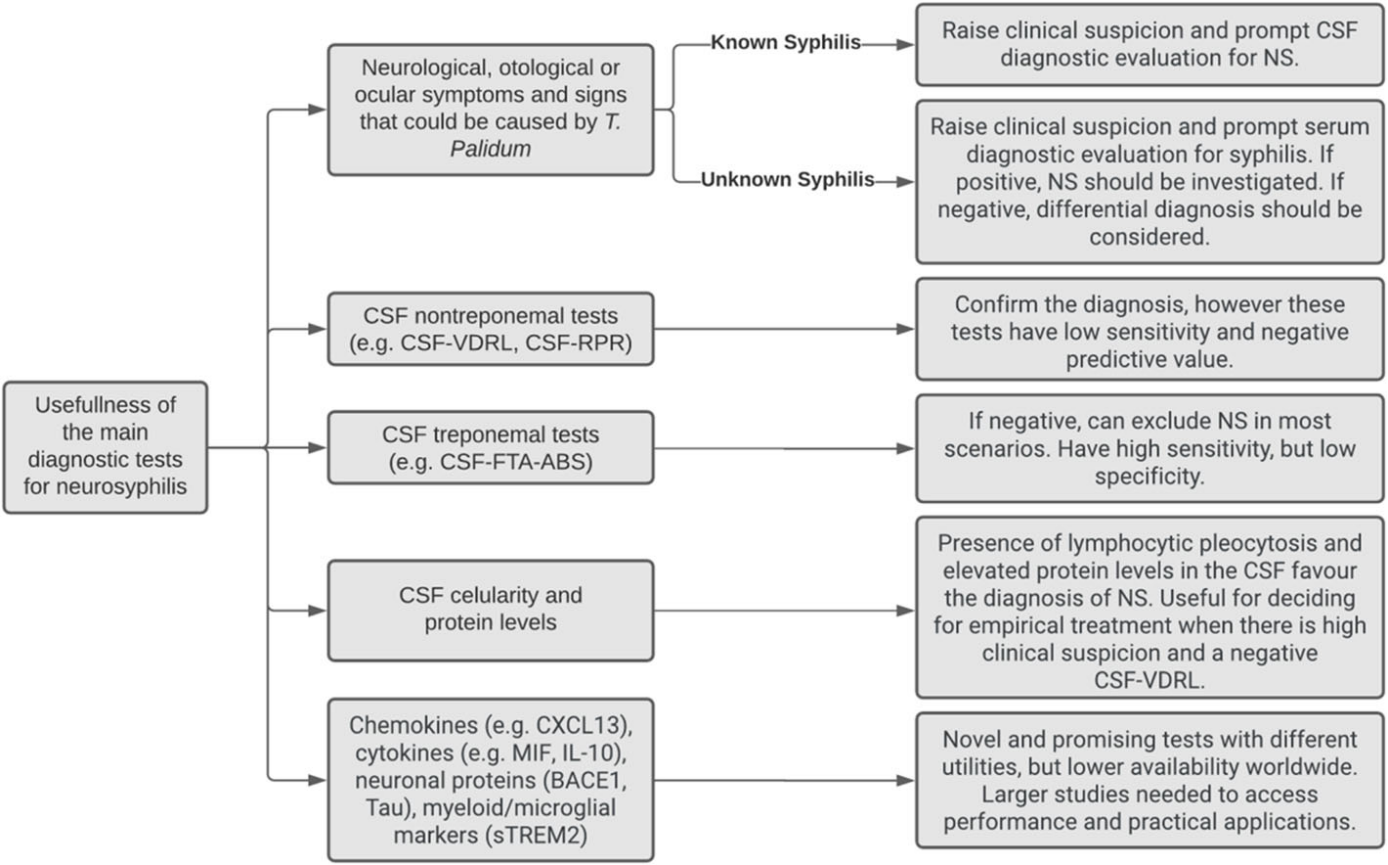
Summary of the main clinical uses of diagnostic methods for neurosyphilis. CSF (cerebrospinal fluid); NS (neurosyphilis); VDRL (venereal disease research laboratory); RPR (rapid plasma reagin); FTA-ABS (fluorescent treponemal antibody absorption); CXCL (chemokine CXC ligand); MIF (macrophage migration inhibitory factor); sTREM2 (soluble triggering receptor expressed on myeloid cells 2); BACE1 (beta-site app-cleaving enzyme 1); IL-10 (interleukin 10)

Figure 4 shows a chart that summarizes the main clinical roles of different diagnostic tests for neurosyphilis.

We believe that the main finding of this review was the great diversity of diagnostic criteria to define neurosyphilis. Seven of the 14 articles admitted explicitly the non-existence of a good gold standard. It affected the secondary objective of our study, which was the accuracy assessment. With different gold standards, the sensibility and specificity values vary in such a way that comparisons between tests in different studies are impaired.

Part of this issue stems from a lack of a precise understanding of the pathogenesis of neurosyphilis. Because the maintenance of long-term cultures of *Treponema pallidum* is a difficult technique, very few studies have investigated its interactions with CNS resident cells. There is limited knowledge about how the presence of the bacteria in the CNS is associated with a higher risk of developing active illness - as a transient invasion of the CNS has been reported in asymptomatic patients with positive nontreponemal tests in CSF [30]. Moreover, Single Nucleotide Polymorphisms in genes that transcribe proteins associated with the innate immune response, namely Toll-Like Receptors, are associated with a higher risk of developing neurosyphilis after acquiring syphilis [40]. It demonstrates that the development of NS consists of a complex interaction between the bacterial capacity of invasion of the CNS, evasion of the immune response, and host ability to clear the pathogen effectively.

Our review has limitations that should not be ignored. The most important are the restricted period of search (5 years), the lack of some sample information in the included papers (e. g. HIV status, age, and sex data), and the impossibility of performing statistical analysis or even simple comparisons with the sensibility and specificity values (due to the heterogeneity and discrepancies of the gold standards). Furthermore, it should be considered that methodological failures in the included articles rebound indirectly in our study. There were papers in which the diagnostic test being evaluated was included in the gold standard for NS diagnosis, increasing accuracy. Some studies utilized limited or clinically unapplicable gold standards. Thus, our results of sensibility and specificity exposed in Table 1 should be carefully interpreted, considering the gold standard used and the methodology of the original articles.

## Conclusion

The diagnosis of neurosyphilis is still a challenge for physicians, and, despite the variety of the existing and developing techniques, clinical suspicion plays the main role. The multiplicity of gold standards adopted in the studies reveals the imprecision and the heterogeneity of the current definitions of neurosyphilis and shows that an important next step for the scientific community is to create a universal diagnostic definition for this disease. This would be a first step to be used by clinicians for a better-standardized diagnosis, and by researchers for future assessment of new diagnostic tools.

## Supplementary Information

**Additional file 1.**

**Additional file 2.**

## Data Availability

The datasets used and/or analysed during the current study are available from the corresponding author on reasonable request.

## Abbreviations

CNS: Central nervous system
NS: Neurosyphilis
ANS: Asymptomatic neurosyphilis
NS (+): Positive neurosyphilis diagnosis
NS (−): Negative neurosyphilis diagnosis
HIV: Human immunodeficiency virus
CSF: Cerebrospinal fluid
WBC: White blood cells
VDRL: Venereal disease research laboratory
RPR: Rapid plasma reagin
USR: Unheated serum reagin
TPPA: *T. pallidum* particle agglutination
TPHA: *T. pallidum* hemagglutination
FTA-ABS: Fluorescent treponemal antibody absorption
PCR: Polymerase chain reaction
RT-PCR: Reverse transcriptase polymerase chain reaction
CMV: Cytomegalovirus
CXCL: Chemokine CXC ligand
MIF: Macrophage migration inhibitory factor
sTREM2: Soluble triggering receptor expressed on myeloid cells 2
BACE1: Beta-site app-cleaving enzyme 1
IL10: Interleukin 10
