# Supplementary material for: Diagnostic tools for neurosyphilis: a systematic review
Source: BMC Infect Dis. 2021 Jun 14;21:568. doi: 10.1186/s12879-021-06264-8 (PMC8201870; doi:10.1186/s12879-021-06264-8)
Supplement: Supplementary file 1 — Additional file 1. [file 12879_2021_6264_MOESM1_ESM.docx]

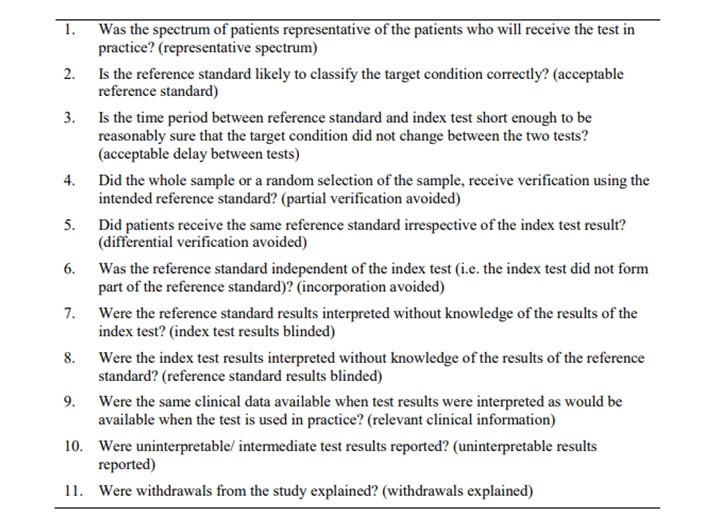


***Supplementary Figure S1.*** *Methodological quality assessment tool endorsed by the Cochrane Handbook for Systematic Reviews of Diagnostic Test Accurac**y* **[13]***.*
