# Supplementary material for: Diagnostic tools for neurosyphilis: a systematic review
Source: BMC Infect Dis. 2021 Jun 14;21:568. doi: 10.1186/s12879-021-06264-8 (PMC8201870; doi:10.1186/s12879-021-06264-8)
Supplement: Supplementary file 2 — Additional file 2. [file 12879_2021_6264_MOESM2_ESM.docx]

| **Table S1: Characteristics of the 14 studies included in the review of diagnostic methods for neurosyphilis** | | | | | | |
| --- | --- | --- | --- | --- | --- | --- |
| **1^st^ Author** | **Year of publication** | **Country** | **Study design** | **Sample sex distribution**  **(% male)** | **Sample age distribution**  **(mean or median, years)** | **Reference** |
| Lu, Y. | 2019 | China | Case-Control | 75% | 47 | 15 |
| Marra, C. | 2017 | USA | Diagnostic Test | 98% | 40 | 16 |
| Merins, V | 2015 | Germany | Diagnostic Test | Not informed | Not informed | 17 |
| Versiani, I. | 2019 | Brazil | Diagnostic Test | 66% | 48 | 18 |
| Ho, E. L. | 2015 | USA | Diagnostic Test | 91% | Not informed | 19 |
| Guarner, J. | 2015 | Canada | Diagnostic Test | 69% | 49 | 20 |
| Xiao, Y. | 2017 | China | Cross-sectional | 70% | 53 | 21 |
| Cai, S. | 2017 | China | Cross-sectional | 35% | 33 | 22 |
| Cecarelli, G. | 2019 | Italy | Cross-sectional | 96% | 46 | 23 |
| Castro, R | 2016 | Portugal | Diagnostic Test | Not informed | “Adults” | 24 |
| Vanhaecke, C. | 2016 | France | Diagnostic Test | 90% | 46 | 25 |
| Wang, C. | 2016 | China | Diagnostic Test | 67% | 51 | 26 |
| Mothapo, K. | 2015 | Netherlands | Diagnostic Test | 90% | 44* | 27 |
| Zeng, Y. | 2016 | China | Diagnostic Test | 60% | 45 | 28 |

***Supplementary Table S1.*** *Characteristics of the 14 studies included in the review of diagnostic methods for neurosyphilis.*
